# Expression of concern: LncRNA CASC2 inhibits autophagy and promotes apoptosis in non-small cell lung cancer cells *via* regulating the miR-214/TRIM16 axis

**DOI:** 10.1039/d2ra90006h

**Published:** 2022-02-16

**Authors:** Laura Fisher

**Affiliations:** Royal Society of Chemistry Thomas Graham House, Science Park, Milton Road Cambridge UK CB4 0WF, UK advances-rsc@rsc.org

## Abstract

Expression of concern for ‘LncRNA CASC2 inhibits autophagy and promotes apoptosis in non-small cell lung cancer cells *via* regulating the miR-214/TRIM16 axis’ by Qian Li *et al.*, *RSC Adv.*, 2018, **8**, 40846–40855, DOI: 10.1039/c8ra09573f.

*RSC Advances* is publishing this expression of concern in order to alert readers that concerns have been raised over the integrity of the data published in this article. The authors have been contacted but have not responded to requests to provide raw data. An expression of concern will continue to be associated with the article until a conclusive outcome is reached.

Laura Fisher, Executive Editor, *RSC Advances*

Date: 31st January 2022

## Supplementary Material

